# The Incidence of Non-Tuberculous Mycobacteria in Infants in Kenya

**DOI:** 10.1155/2019/1273235

**Published:** 2019-07-03

**Authors:** Grace Kaguthi, Videlis Nduba, Wilfred Murithi, Suzanne Verver

**Affiliations:** ^1^Kenya Medical Research Institute-Centre for Respiratory Diseases Research, Nairobi, Kenya; ^2^Amsterdam University Medical Centre, University of Amsterdam, Netherlands; ^3^Department of Public Health, Erasmus Medical Centre, Rotterdam, Netherlands; ^4^KNCV Tuberculosis Foundation, The Hague, Netherlands

## Abstract

There is inadequate understanding of the epidemiology of Non-Tuberculous Mycobacteria (NTM) among infants in high tuberculosis burden countries. The objective of this study was to document the incidence and diversity of NTM disease or colonisation in sputum specimens from infants with presumptive TB, the risk factors, and clinical characteristics, in a high TB and HIV burden setting in Western Kenya. A cohort of 2900 newborns was followed for 1–2 years to assess TB incidence. TB investigations included collection of induced sputa and gastric aspirates for culture and speciation by HAIN®, Tuberculin Skin Testing (TST), HIV testing, and chest radiography. The American Thoracic Society Criteria (ATS) were applied to identify NTM disease. Among 927 (32% of 2900) with presumptive TB, 742 (80%) were investigated. NTM were isolated from 19/742 (2.6%) infants.* M. fortuitum* was most frequently speciated (32%). Total person-time was 3330 years. NTM incidence was 5.7/1,000 person-years, 95% CI (3.5, 8.7). Infants diagnosed with TB were more likely to have NTM isolation (odds ratio 11.5; 95% CI 3.25, 41.0). None of the infants with NTM isolated met the criteria for NTM disease. The incidence of NTM isolation was comparable to similar studies in Africa. NTM isolation did not meet ATS criteria for disease and could represent colonisation. TB disease appears to be structural lung disease predisposing to NTM colonisation.

## 1. Introduction

Non-Tuberculous Mycobacteria (NTM) are environmental saprophytes widely distributed in water and soil [1]. They are the genetic progenitors of* M. Tuberculosis *Complex (MTBC), after a series of gene deletions and gene acquisitions [2] with MTBC evolving to a more virulent pathogen. NTM rarely cause disease except when immune function is impaired [3], elderly patients and chronic lung disease. However, some NTM are pathogenic, and recently there has been a reported increase in NTM lymphadenitis [4, 5] and Buruli ulcers [6, 7].

The shared ancestry of NTM and MTBC is responsible for immune interference in BCG vaccination, via cross reactive immune responses [2]. This could be one of the reasons for low BCG efficacy where NTM are prevalent [2]. Absence of NTM sensitization was associated with higher efficacy of BCG against pulmonary and severe forms of tuberculosis in a systematic review [8]. Surprisingly, the discontinuation of universal BCG vaccination in these countries has seen an increase of NTM lymphadenitis in children, suggesting BCG was also protecting against NTM in that setting [4]. NTM appear to be immune modulators influencing host interactions in BCG efficacy, TB burden, and NTM disease.

The antigen homologues [2] further decrease accuracy of biomarkers distinguishing latent TB infection (LTBI) and NTM exposure.

Pulmonary NTM disease is clinically and radiologically identical to TB and is so diagnosed, in the absence of microbiological confirmation in high TB burden settings. It is a relevant distinction to make as almost all NTM do not respond to anti-tuberculous therapy [9]. Isolation of NTM in sputum is not necessarily disease [9]. Data on NTM disease and prevalent subtypes is limited particularly in countries with a high TB burden. Most studies report on adults [10–12]. Few studies on NTM in children have been published on the continent [13–15]. Most document the proportion of NTM among those with presumptive TB. There is also a dearth of knowledge on risk and exposure factors. As infants are the target age group for TB vaccines in the pipeline, it is useful to describe the epidemiological landscape of NTM, given their role in tuberculosis incidence and possibly vaccine efficacy.

The objective of this study was to document the incidence and diversity of NTM disease or colonisation in sputum specimens from infants with presumptive TB, the risk factors, and clinical characteristics, in a high TB and HIV burden setting.

## 2. Study Population and Methods

The study took place in Siaya, Western Kenya, a predominantly rural community north of Lake Victoria. The area has a high prevalence of HIV, TB, and malaria. Most women delivered at home [21]. The NTM substudy was part of a prospective cohort study to document the incidence of TB ahead of TB vaccine trials in the same population. Presumably, infants are born uninfected; we present the incidence of NTM in this cohort.

Briefly, parents or guardians of 2900 infants aged zero to six weeks gave written permission for enrollment of their newborns between June 2009 and June 2010. Patients were followed up for at least one year and a maximum of two years. Through four monthly scheduled visits and ancillary care visits, infants were identified as having presumptive TB if they had history of TB contact, symptoms, or signs of pulmonary TB (failure to thrive, cough or night sweats or fever for more than two weeks, a history of hospitalization for HIV/AIDS related illness, lower respiratory tract infections, meningitis, or TB). Consequently, they were admitted into a case verification ward for three days. Two fasted sputum induction specimens and two gastric aspirates were collected on subsequent mornings. Tuberculin Skin Testing (TST) was done with two Tuberculin Units (2TU) from Statens Serum Institut (SSI). TST readings of 10mm and more or 5mm or more among HIV infected children were considered to be positive readings. Further, DNA PCR HIV (COBAS® HIV-1 Amplicor by ROCHE) tests and digital chest radiography were performed.

Patients received anti-tuberculous therapy if they had microbiological confirmation (definite TB) or clinically, based on the Keith Edward TB Score (KE Score) Chart of >7, or <7 if the chest radiograph was suggestive (probable TB). Mid-Upper Arm Circumference (MUAC) was used to determine nutritional status for children older than 6 months old at time of TB investigations. Weight for Age Z Score was used for those less than 6 months. HIV infected infants were referred for anti-retroviral treatment initiation and care. Patients vital status at last study contact was documented.

Chest radiographs were read systematically and classified as abnormal probable TB, abnormal not TB, or normal [22]. The study was approved by Kenya Medical Research Institute Independent Ethics Committee (KEMRI-IEC) SSC 1465. The data used to support the findings of this study are available from the corresponding author upon request.

We applied the American Thoracic Society's [23] criteria to establish clinical significance of positive NTM cultures.

### 2.1. Laboratory Methods and Sample Decontamination

Induced sputum and gastric aspirates were transported to the laboratory at 2 to 8°C, processed using freshly prepared N-acetyl L-cysteine (NALC)-4% sodium hydroxide (NaOH)-2.9% sodium citrate at a final concentration of 1%. Gastric aspirates with >5ml volume were concentrated by centrifugation and pellet resuspended with 5ml phosphate buffer saline (PBS). Digestion was stopped using pH 6.8 PBS after 20 minutes. Centrifugation was done at 3,000 x g for 15 minutes at 4°c. Supernatant was discarded and the pellet resuspended with 2ml PBS. This was used for inoculation of Lowenstein Jensen (LJ) [BD] media (0,2ml), fluorescent microscopy, and mycobacteria growth indicator tube (MGIT) [BD] (0.5ml). LJ were incubated in 37°C CO_2_ incubators for 8 weeks, and MGIT was incubated in automated BACTEC ™ MGIT ™ 960 [BD] for 42 days. Artificial sputum was used as a negative control sample to check for cross-contamination with each batch processed.

MGIT cultures that turned positive were stained for acid fast bacilli (AFB) using Ziehl Neelsen (ZN). Contamination was checked by inoculation and incubation of blood agar plates at 37°c and read after 48 hours. Samples that tested ZN negative but Blood Agar Plate (BAP) positive ≥7 days later were discarded as contaminated. Those <7 days were redigested using 4% NaOH as described in MGIT™ procedure manual [24]. AFB positive cultures were tested by immunochromatographic assay (ICA) such as Capilia™ TB-Neo (TAUNS Laboratories, Numazu, Japan) or BD MGIT™ TBc identification kit ((BD, Franklin Lakes, NJ, USA) to identify whether NTM or MTBC.

For LJ cultures with visible growth, we assessed colony morphology. Those suggestive of mycobacteria were identified using ZN smear, and those AFB positive were tested with ICA.

NTM culture isolates were genetically identified to the species level using Genotype Mycobacterium Common Mycobacterium (CM) or Additional Species (AS) kits (HAIN Lifescience, Nehren, Germany). The procedure was done according to manufacturer's instructions.

### 2.2. Statistical Methods

Frequency methods were used to describe the baseline characteristics. Odds ratios were used to analyze whether differences between those with and without NTM were due to chance. T-tests were used to compare the mean age at TB investigations. To evaluate differences in clinical characteristics, known and potential risk factors, logistic regression was performed. NTM cases that had microbiologically confirmed or clinical TB were analysed as TB cases. A-priori risk factors included infant and maternal HIV infection, nutritional status, housing, and number of siblings.

## 3. Results

**Figure 1:**
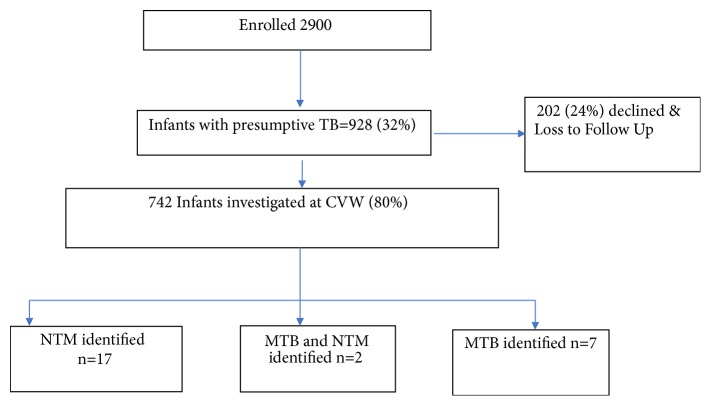
Study flow chart.

Of 2900 infants enrolled, 927 (32%) were suspected to have TB (presumptive TB) during their 1-2-year follow-up. Of these 742 (80%) were admitted for investigations (Figure 1). There were 19 NTM identified following culture (2.6% of 742). Total person-time of follow-up was 3330.3 years. The incidence of NTM was 5.7 per 1,000 person-years (pyo) of follow-up (95% CI 3.5, 8.7), while all TB incidence (49 cases) was 15/1000 pyo (95% CI, 11-20) and microbiologically confirmed TB incidence was 2.7/1,000 pyo. At baseline, there were no statistically significant differences between those who had NTM identified versus all other infants (Table 1).

Upon bivariate comparison of clinical characteristics between presumptive TB patients and NTM cases, there were no statistically significant differences (Table 2(a)). However, odds of a positive NTM among infants with TB was eleven-fold that of infants with no TB (OR 11.6 (95% CI 3.25, 41.0). NTM cases had forty-eight-fold higher odds of having microbiologically confirmed TB compared to all presumptive TB (OR 48.3 95% CI 9.3, 249) (Table 2(a)).

There were no differences between NTM cases and other presumptive TB cases in mean age at time of TB investigations (Table 2(b)).

Table 3 shows the NTM identified and the individual's clinical characteristics.* M. fortuitum* (6/19 32%) and* M. scrofulaceum* (2/19 11%) were most frequently isolated. Two of the 19 (11%) were unidentifiable. Two patients had MTBC and NTM coinfection.

Applying the ATS criteria for diagnosis of NTM disease, none of the NTM cases qualified as having NTM disease. Only 1/19 (5.3%) NTM case was HIV infected which had NTM cultured (*M. asiaticum*) while 3/19 (16%) were born to mothers who tested HIV positive but were themselves uninfected (HUE).

In our study, rapidly growing mycobacteria (RGM), which form colonies in less than seven days, were isolated most frequently (10/19) (Table 3). The most frequently isolated NTM in pediatric studies are shown in Table 4.* M. fortuitum* was the most frequently isolated NTM among the identified studies.

## 4. Discussion

### 4.1. Burden of NTM

The proportion of NTM in pulmonary samples of presumptive TB cases in this infant cohort was relatively low (2.6%; 95% CI 1.5, 3.8). Standard sputum decontamination procedures were judiciously applied; hence it is unlikely that NTM yield was affected by this. A similar study among infants in Uganda and South Africa found 3.7% [14] and 6% [13], respectively. The epidemiology of exposure in this region could be nonlinear, where exposure in early childhood is minimal but increases rapidly in adolescents. A significantly higher proportion of NTM were identified among presumptive TB cases in adolescents in the study area (37.5%), at the time of the study (V. Nduba, Personal Communication). Nevertheless, the Mozambique cohort and a survey in Ethiopia had more NTM [15, 20], and the average prevalence in African adult pulmonary samples was 7.5% in a systematic review [10]. It is possible that BCG is protective against NTM colonisation. A twenty-year retrospective study of NTM notifications in children demonstrated increased odds of NTM disease when universal BCG vaccination was halted in Finland [4]. Therefore, BCG could also protect against colonisation. This can be evaluated conclusively in head to head comparisons of BCG and recombinant BCG vaccines presently in phase III clinical trials [25].

### 4.2. Colonisation or NTM Disease/Clinical Relevance

We did not find statistically significant differences in baseline characteristics between NTM cases and other presumptive TB patients suggesting widespread exposure across the study population. There were no differences in the clinical or radiological characteristics between presumptive TB and NTM cases.

NTM disease is clinically and radiologically indistinguishable from TB [9]. Two NTM cases were symptomatic with a suggestive radiological picture and would have qualified as NTM disease, but MTBC was also isolated from their sputum. The remainder had no combination of suggestive clinical or radiological features. We therefore conclude the NTM cases represent colonization. There is a possibility that these are laboratory contaminants; however this is unlikely since we checked for contaminants by having negative controls.

### 4.3. Risk Factors

#### 4.3.1. Environmental Exposure

We did not identify any environmental risk factors for NTM incidence. Unlike MTBC which is transmitted from person to person, NTM transmission occurs via repeated environmental exposure. In infants, this would be through handling by parents and siblings. The study area is rural. Risk for acquiring NTM is significantly higher in communities engaged in occupations that generate aerosols and are exposed to soil for prolonged periods such as agriculture [26]. It is not clear what the environmental source of these NTM is.

### 4.4. Host Factors

Host factors predisposing to NTM isolation were intercurrent MTBC disease and severe undernutrition, although the latter did not reach statistical significance. Past history of TB has been known to be a risk factor for NTM disease [27, 28], since we studied infants that could not be confirmed. Interestingly, in this study, MTBC isolation increased the odds of NTM isolation almost fifty-fold. NTM-MTBC coinfection in the same infant host has been observed [13, 15], and in adults in high TB burden countries [29]. TB appears to be a preexisting lung condition predisposing to NTM colonisation [9].

Low Body Mass Index and poor nutrition are other possible host factors, even predicting risk of disseminated NTM disease in other studies [30, 31], our study seemed to show the same trend.

Only in one case was the NTM case HIV infected, indicating among infants in this region, immunodeficiency is not a factor in NTM isolation in sputum.

### 4.5. NTM Isolated

The spectrum of organisms identified in this NTM study is similar in type and frequency to those reported in Uganda [14], Ethiopia [20], and Saudi Arabia [28].* M. fortuitum* was most frequently isolated in these studies. There could be geographic and climatic factors in the distribution. All the regions have warm climates. Increase in latitude and polarity has been shown to be associated with higher isolation rates of more pathogenic, slow growing mycobacteria [4, 13, 15].

### 4.6. TB Diagnostics

There was no detectable difference in TST positivity between NTM cases and other patients whereas NTM sensitization is known to be responsible for false positive TST readings. Indeed false positive TSTs due to NTM are infrequent and mainly relevant in areas with low TB endemicity [32].

### 4.7. BCG Efficacy

NTM influence the relative efficacy of BCG vaccines [33]. The nature and type of NTM isolated in TB endemic countries are critical to an efficient vaccination campaign [2]. The relative frequency of isolated species may correlate with the prevalence of skin sensitivity to their antigens, as was shown in Malawi [11]. RGMs have been shown to be protective against leprosy and TB [11]. This could not be confirmed in the current study due to the low numbers of NTM isolated.

As there was no unvaccinated control group, it is not possible to assess efficacy of BCG. Thus, it appears that the risk of exposure to NTM as a covariate of vaccine efficacy, as has been previously suggested, is quite low in the target age group.

### 4.8. Limitations

Our analysis was limited due to the small proportion of NTM isolated in this age group. Nevertheless, it forms a baseline assessment for future studies including future vaccine trials.

Also, not all infants could be tested for NTM; this was not the primary objective, and it is challenging to obtain samples from children without presumptive TB. Therefore, the NTM incidence may be an underestimate of the NTM burden in the population.

## 5. Conclusions

This study has attempted to document the incidence of NTM among infants thought to have TB. The clinical relevance of NTM isolated points to colonisation and not disease, as all the infants from whom NTM were isolated did not meet the ATS criteria for disease. Our data shows that a patient presenting with features of TB is less likely to have NTM disease, in similar settings.

## Figures and Tables

**Table 1:** Baseline characteristics of study sample, infants with presumptive TB and infants with NTM isolated; and comparison between infants with presumptive TB with and without NTM.

Characteristic	Study Sample (n=2900)	Investigated for Presumptive TB (n =742) (N, column %)	NTM positive (n=19) (N, row%)	OR (95%CI) *∗*
*Gender*				
Female	1412	358 (48%)	10 (2.8%)	1 (ref)
Male	1488	384 (52%)	9 (2.3%)	0.85 (0.35, 2.11)

*Enrolment weight*				
Normal	2674	667 (90%)	16 (2.4%)	1 (ref)
low	226	75 (10%)	3 (4.1%)	2.24 (0.65, 7.73)

*Place of birth*				
Home	1840	510 (69%)	11 (2.2%)	0.77 (0.31, 1.93)
Health facility	1038	229 (31%)	8 (3.5%)	1 (ref)
missing	22	3 (<1%)		

*Maternal HIV status*				
HIV negative	2451	598 (81%)	16 (2.7%)	1 (ref)
HIV positive	401	127 (17%)	3 (2.4%)	0.88 (0.25, 3.08)
Unknown	48	17 ( 2%)		

*Infant HIV status*				
HIV negative	2827	708 (95%)	18 (2.5%)	1 (ref)
HIV positive	73	34 (5%)	1 (2.9%)	2.17 (0.29, 16.5)

*Maternal age category*				
<19	635	152 (21%)	1 (0.7%)	1 (ref)
20-29	1533	384 (52%)	16 (4.2%)	6.69 (0.89, 50.5)
>29	732	206 (28%)	2 (1.0%)	1.74 (0.16, 19.2)

*Maternal Occupation*				
Unemployed	1676	409 (55%)	11 (2.7%)	1 (ref)
Farmer	864	250 (34%)	5 (2.0%)	0.88 (0.31, 2.54)
Business	260	61 (8%)	2 (3.3%)	1.17 (0.26, 5.32)
Salaried	71	13 (2%)	1 (7.7%)	2.16 (0.28, 17.0)
Unknown	29	9 (1%)		

*Housing Type*				
Mud House	1912	523 (71%)	11 (2.1%)	1(ref)
Semi-permanent	527	125 (17%)	4 (3.2%)	1.32 (0.42, 4.17)
Permanent	426	84 (11%)	4 (4.8%)	1.64 (0.52, 5.17)
Other	6	1 (0.1%)		
Unknown	29	9 (1.2%)		

*Number of Siblings*				
None	649	129 (17%)	3 (2.3%)	1 (ref)
One to three	1497	391 (53%)	14 (3.6%)	2.03 (0.58, 7.10)
>3	754	222 (30%)	2 (0.9%)	0.57 (0.10, 3.44)

*Vaccination Status at 6 weeks*				
Complete	2205	682 (92%)	16(2.4%)	0.48 (0.11, 2.10)
Incomplete	133	29 (4%)	2(7.0%)	1 (ref)
Missing	562	31 (4%)	1(3.2%)	

*∗* Odds of being NTM case among those investigated for presumptive TB, given the category of baseline characteristic.

**Table:** (a) Comparative clinical characteristics of those investigated for presumptive TB and infants with NTM isolated (categorical).

Clinical Characteristics	Presumptive TB	NTM +ve	OR (95%CI)
	N (column %)	N (row%)	
	(n=742)	(n=19)	
*Any TB case (clinical or confirmed)*			
No	694 (94%)	16 (2.3%)	1 (ref)
Yes	48 (6.5%)	3 (6.3%)	11.6 (3.25, 41.0)

*MTBC +ve TB case*			
No	733 (99%)	17 (2.3%)	1 (ref)
Yes	9 (1%)	2 (22.2%)	48.3 (9.34, 249)

*Chest Radiograph*			
Normal	590 (80%)	13 (2.2%)	1(ref)
Abnormal not TB	110 (15%)	4 (3.6%)	1.71 (0.55, 5.35)
Abnormal TB	35 (5.0%)	2 (5.7%)	2.80 (0.61, 12.9)
missing	7 (0.9%)		

*Keith Edward TB score*			
<7	675 (90%)	17 (2.5%)	1 (ref)
>=7	32 (4.3%)	2 (6.3%)	2.62 (0.58, 11.9)
Missing	35 (4.7%)		

*Reason for TB suspicion*			
History of hospitalization			
No	283 (38%)	8 (3.4%)	1 (ref)
Yes	426 (57%)	11(2.6%)	0.78 (0.31, 1.97)
Missing	33 (5.0%)		

*TB Contact History*			
No	579 (78%)	13(2.3%)	1 (ref)
Yes	131 (18%)	6 (4.6%)	2.57 (0.96, 6.88)
Missing	32 (4.0%)		

*TST results*			
Negative	555 (76%)	14 (74%)	1 (ref)
Positive	172 (24%)	5 (26%)	1.15 (0.41, 3.25)

*TB symptoms*			
No	530 (71%)	15 (2.8%)	1 (ref)
Yes	180 (24%)	4 (2.2%)	0.85 (0.28, 2.58)
Missing	32 (4.0%)		

*Nutritional Status at admission*			
Healthy	379 (51%)	8 (2.1%)	1 (ref)
At risk	195 (27%)	6 (3.0%)	1.48 (0.51, 4.32)
Moderate Acute	113 (15%)	2 (1.8%)	0.84 (0.18, 4.03)
Malnutrition (MAM)			
Severe Acute	40 (5%)	3 (7.5%)	3.73 (0.95, 14.7)
Malnutrition (SAM)			
Missing	15 (2%)		

**Table:** (b) Comparative clinical characteristics for those investigated for presumptive TB and infants with NTM isolated (continuous variable).

Clinical Characteristic	Categories	n	Mean age (95% CI)	Rank sum p- value/t-test p value
Mean age at TB investigation (months)	NTM negative Presumptive TB	718	9.34 (8.95, 9.74)	0.20

	NTM case	19	11.0 (8.02, 13.9)	

	Missing	5		

**Table 3:** NTM identified; clinical and radiological profile of cases.

Number		Age (months) at admission	NTM species	MTBC +ve	Infant HIV status	Nutritional status at admission	TST reading (mm)	KE Score	Vital Status	CXR	Siblings	Housing
*Rapidly Growing Mycobacteria*												

1	52452	14	*M. peregrinum*	No	Negative	At risk	0	0	alive	Normal	4	Mud

2	50170	5	*M. smegmatis*	No	Negative	Healthy	4	0	alive	Normal	1	Semi

3	50220	11	*M. smegmatis*	No	Negative	At risk	3	1	alive	Normal	unknown	Semi-

4	51388	5	*M. chelonae*	No	Negative	Healthy	0	0	Alive	Normal	2	mud

5	52696	5	*M. fortuitum1*	No	Negative	Healthy	0	0	Alive	Abnormal not TB	6	mud

6	52727	13	*M. fortuitum1*	No	Negative	SAM	10	6	alive	Normal	3	mud

7	50206	6	*M. fortuitum1*	No	Negative	Healthy	12	3	Alive	Normal	1	stone

8	50523	22	*M. fortuitum1*	No	Negative	At risk	0	0	Alive	Normal	1	stone

9	51104	19	*M. fortuitum1*	No	Negative	Healthy	1	1	Alive	Normal	1	mud

10	52024	7	*M. fortuitum2*	No	Negative-HUE	Healthy	0	1	alive	Normal	1	mud

*Slow Growing Mycobacteria*												

11	51599	9	*M. asiaticum*	Yes	Positive	SAM	7	10	Died	Abnormal not TB	unk	stone

12	50049	4	*M. celatum*	No	Negative	MAM	0	0	Alive	Normal	3	mud

13	51598	7	*M. gordonae*	No	Negative	At risk	1	1	alive	Abnormal not TB	3	mud

14	52683	9	*M. intracellulare*	No	Negative	Healthy	0	0	alive	Abnormal not TB	3	Semi-

15	51119	12	*M. malmoense*	No	Negative	At risk	2	1	alive	Normal	2	Semi-

16	50380	23	*M. scrofulaceum*	No	Negative-HUE	At risk	12	4	alive	Abnormal TB likely	3	Mud

17	50108	20	*M. scrofulaceum*	No	Negative	Healthy	3	3	alive	Normal	1	mud

*Unidentified Mycobacteria*												

18	50178	11	Unidentified	No	Negative-HUE	Healthy	0	0	alive	Normal	unk	mud

19	51706	6	Unidentified	Yes	Negative	SAM	12	13	alive	Abnormal TB likely	2	stone

**Table 4:** Pediatric NTM studies in Africa between years 2000 and 2018.

Authors, Country, Year of Publication	Study Type	Study Population	NTM proportion of Presumptive TB	Most frequently isolated NTM	Clinical Relevance*∗*	MTBC-NTM co-infection	Proportion of participants with TB	National/local TB prevalence per 100, 000 at time of study
Present Study (Kenya)	Prospective Cohort Study	<2 years	2.6%	*M. fortuitum* (32%)	Colonisation	2/19	1.5%	600 [16]

Asiimwe B, Uganda 2013 [14]	Prospective Cohort Study	<1 year	3.7%	*M. fortuitum* (64%)	Not specified	0	Not specified	193 [17]

Hatherill M, South Africa 2006 [13]	Prospective Cohort Study	<2 years	6%	*M. intracellulare* (41%)	7/109-NTM disease	5/109	11%	960 [18]

Lopez-Varela E, Mozambique 2017 [15]	Prospective Cohort Study	<2 years	26%	*M. intracellulare* (68%)	Colonisation	0	>1.4%	>544 [19]

Workalemahu B, Ethiopia 2013 [20]	Cross sectional Hospital Survey	<15 years	9.9%	*M. fortuitum *(29%)	Not specified	0	15%	237 [20]

*∗*Based on authors' description of suggestive clinical and radiological features.

## Data Availability

The data used to support the findings of this study are available from the corresponding author upon request.
